# 

**Published:** 1890-08-23

**Authors:** 


### NOTICE TO CORRESPONDENTS.

All MS., Letters, Books for Review, and other matters intended for the Editor should be addressed The Editor, The Lodge, Porchester Square, London, W.

The Editor cannot undertake to return rejected M.S., even when accompanied by stamped directed envelope.

**Notice to Advertisers and Subscribers.**

All Advertisements, Orders for Copies of The Hospital, and Business Communications should be addressed to The Publisher, *not to the Editor*, The Hospital, 140, Strand, W.C.

**Bound Volumes of "The Hospital."**

Vol. VI., containing full page portraits of T.R.H. the Prince and Princess of Wales, and Miss Florence Nightingale, is obtainable at 4s., post free.

Orders will be received for Vol. VII., 4s. post free. Cases for binding may be had of the Publisher, at 1s. 6d. each.

**To Residents, Secretaries, and Matrons.**

The Editor will be glad to have the Regulations and each Report as issued by Asylums, Hospitals, Convalescent and Nursing Institutions, as well as those of other Charities, and an early copy in duplicate of all Appeals and Festival Notices, etc., addressed to *The Editor, The Lodge, Porchester Square, London, W.* Any superintendent, secretary, matron, or other chief official who regularly sends such information may be registered, on application to the Publisher, to receive free of cost a cover for binding The Hospital in half-yearly volumes.

### BURDETT'S HOSPITAL ANNUAL FOR 1890

Is now ready, and may be obtained of the Publisher, The Hospital Offices, 140, Strand, W.C., price 2s. 6d. limp boards, or 3s. 6d. cloth. All orders must be accompanied by a remittance.

### GENERAL CHARITIES.

[This Column is devoted to an account of work of charitable institutions other than Medical Charities. The Editor will be glad to receive reports or news of work at 140, Strand. *Philanthropy* should be plainly written on the envelope.]

Fourteen years ago the Rev. W. D. Maclagan, now Bishop of Lichfield, started a fund which was to enable poor clergy to take a holiday from their work, when without assistance this would be an impossibility. There is no man works harder than a conscientious London clergyman; he is always amidst poverty and sorrow, often in unhealthy atmosphere, and if anybody needs stimulating by fresh scenes, he most certainly does. We are glad to note that last year the **POOR CLERGY HOLIDAY FUND** gave 122 grants compared to 98 of the previous year. The names of the recipients of the fund are not published, which seems to us to add to the value of the gift. The archdeacons assign all grants which they, from personal knowledge of the clergy, are able to do efficiently. All accounts are published annually in the *Guardian*, and there are no office expenses. Any donations will be received with much gratitude by the Rev. Arthur J. Ingram (treasurer), St. Margaret's Rectory, 20, Finsbury Square, E.C.

The cry of the **OVERWORKED GIRLS AND BOYS SERVING IN SHOPS** is nothing new, but it is not the less pitiable and justifiable. We hear every now and again of voluntary efforts made by the shopkeepers to shorten the long hours of standing, and to give a few short hours of rest one evening in the week, then the scheme breaks down, because while one man closes his shop another thinks it's a chance of doing a little extra business, and keeps his open. The struggle for existence is, no doubt, hard, but to exist on "human creatures' lives" is actually what many of the London shopkeepers are doing, some of them, perhaps, through carelessness, others because money they will have, even though it be at such a price. This is not a question for any party politics, it is purely a question of humanitarianism. The factory children and women hands are protected, why are the shop hands left to the tender mercy of employers? Twelve to fifteen hours a-day young girls stand behind the counters always expected to have a pleasant smile for their customers, though often so tired and exhausted they hardly know how to stand up at all. When we hear of the way in which the assistants in less important shops are hunted over their meals, crowded at night into bed-rooms not nearly big enough for the number, working from about seventy to ninety hours of the week in one weary treadmill, it seems very small wonder that when released at night they try to drown their weariness and hopelessness in drink, or are tempted to get their living in some dishonest manner. Is it a marvel, either, that many of them girls and young women especially, fall victims to weakness, illness, and finally to death? Contrast with this the arrangements in the large business houses where the employers are human, and where every effort is made to make the sanitary and social condition of the assistants all it should be—short hours, and a weekly half-holiday—what more could any one want? Public opinion ought to condemn the shops which are unmerciful, but then the public loves a bargain, and very often knows little of the misery and hardship of overwork in shops. If people would only be less selfish, and consult other interests than their own, legislation would often be needless; but till the public is less apathetic than at present, something must be done to help those who cannot help themselves. Mr. Sutherst, who, with Sir John Lubbock, has worked so hard in the question, writes to us most kindly from Dr. Johnson's Buildings, Temple, and we recommend those who want to know more to get his book, "Death and Disease," published by Kegan Paul, Trench, and Co. It may stir people to do their shopping at reasonable hours, induce them to avoid purchasing during the dinner hour, and convince them that "great bargains" are often made through the sacrifice of the lives of overworked and underpaid "shop assistants."

### STUDENTS' COLUMN.

### UNIVERSITY OF LONDON.

### INTERMEDIATE EXAMINATIONS IN ARTS AND IN SCIENCE AND PRELIMINARY SCIENTIFIC (M.B.) EXAMINATION, 1890.

EXAMINATIONS FOR HONOURS.

INTERMEDIATE ARTS ONLY.

LATIN.—*First Class*: Wilkins, Roland Field (Exhibition), Sedbergh School and Owens College; *Goodier, Alban, Stonyhurst College; Gray, Edward Dundas McQ., private study. *Second Class*: Donlevy, James Celestine, Mt. St. Mary's College, Chesterfield; Bullen, Joseph, St. Francis Xavier's College, Liverpool; ‡Almond, John Swarbrick, St. Edward's College, Liverpool; ‡Gardner, Thomas Ignatius, Mt. St. Mary's College, Chesterfield; Crump, Ernest Henry, private study. *Third Class*: ‡O'Ferrall, John G. A. M. J. M., Stonyhurst College; ‡Tomlinson, Christopher Ernest, private study.

ENGLISH.—*First Class*: Thomas, Diana Jane (Exhibition), University College, Cardiff; *Tipping, Llewellyn, private study; *Brockington, Alfred Allen, Mason College and King Edward Grammar School, Aston. *Second Class*: Barrows, Maude Marion, Mason College; Partridge, Jane Bella, Bedford College, London, and University College, Aberystwith; Wall, Arnold, private study; Bevis, James Frederick, private study; Jenkyn-Brown, Lilian E., Mason College; ‡Eastham, Robt., St. Edward's College, Liverpool; ‡Watt, Rebecca Wilson, Bedford College, London; Williams, William Owen, Kingswood School; ‡Allen, Arthur Ormiston, private study; ‡Lee, Herbert, King's College; ‡Trenerry, Charles Farley, Manor House, Clapham, and private study; Webber, Etienne Auguste, private study. *Third Class*: Bateman, Thomas Somerset, Mason College and private study; Murray, Samuel Macculloch, private study; Parkinson, John Harold, Halstead Grammar School; Hurst, Fred, the Old Grammar School, Pontefract; ‡Tomlinson, Christopher Ernest, private study; ‡Urwin, Wilfred Barwick, Bede College, Durham, and private study; Jenkins, William James, University College, Aberystwith; ‡Austin, John Worsley, Mason College; ‡Jones, Frederick, University College, Bangor; §Crump, Ernest Henry, private study; §Walsh, Frederick E. A. Philip, Mercers' School and private study; Shaer, Isidore, Jews' Free School; Cotter, Herbert Robert George, Firth College; Gray, Edward Dundas McQ., private study.

FRENCH.—*First Class*: Stoer, Margaretha, L. (disqualified by age for the prize), University College and private tuition; Gray, Edward Dundas McQ. (disqualified by age for the prize), private study; Partridge, Jane B. (prize), Bedford College, London, and University College, Aberystwith; Daniels, Sidney R., Wycliffe College, Stonehouse. *Second Class*: Theobald, Bertram Gordon, private tuition; Davies, Charles George, private study; Grant, William, private study; Eastham, Robert, St. Edward's College, Liverpool. *Third Class*: Scharlieb, Mary Ethel S., University College and private tuition; Jenkyn-Brown, Lilian E., Mason College; Davidson, Ernest Robert W., Belle Vue House, Norwich, and private study; Bryant, Alfred Charles, King's College.

GERMAN.—*First Class*: Gray, Edward Dundas McQ. (disqualified by age for the prize), private study; Brugger, Ernest (disqualified by age for the prize), University of Strassburg and private study. *Second Class*: Davidson, Ernest Robert W., Belle Vue House, Norwich, and private study; Farrer, Ethel Winifred, Bedford College, London; Clapham, Edith Honor, Bedford College, London. *Third Class*: Watt, Rebecca Wilson, Bedford College, London.

INTERMEDIATE ARTS AND INTERMEDIATE SCIENCE CONJOINTLY.

MATHEMATICS.—*First Class*: McAulay, F. S., Int. Sc (disqualified by age for the Exhibition), private tuition; Franks, Robert S., Int. Arts, St. John's College, Cambridge; Twentyman, J. M., Int. Sc., Christ's College, Cambridge. *Second Class*: Andrade, Samuel, Int. Arts, St. Catherine College, Cambridge, and private study

INTERMEDIATE SCIENCE AND PRELIMINARY SCIENTIFIC (M.B.) CONJOINTLY.

INORGANIC CHEMISTRY.—*First Class*: Bremridge, R. H., Int. Sc. (exhibition), Magdalen College, Oxford; White, Edmund, Int. Sc., private study; *Second Clas*: Edmunds, E. W., Int. Sc., King Edward High School and Mason College, Birmingham; †Warrington, R. J., Pr. Sci., High School, Newcastle-under-Lyme, and University College, Aberystwith; †Hora, Julian, Prel. Sci., Guy's Hospital; Thompson, G. R., Int. Sc., Yorkshire College, Science Schools, South Kensington, and private study; Fournier d'Albe, E. E., I. S., Normal School of Science and private study; Barker, A. H., B.A., Int. Sc., University College, Aberystwith. *Third Class*: Weston, F. Edwin, Int. Sc., private study; Franck, C. E., Prel. Sci., University College; Blair, Robert, Int. Sc., Bromley School of Science, King's College, and private study; Thomas, G. Lester, Int. Sc., University College, Bristol; Eumorfopoulos, N., I. S., University College.

EXPERIMENTAL PHYSICS.—*First Class*: ‡Holt, Jane B., Int. Sc. (Disqualified by age for the Neil Arnott Exhibition and Medal), University College, Liverpool; ‡Thompson, G. R., Int. Sc. (Neil Arnott Exhibition and Medal), Yorkshire College, Science School, South Kensington, and private study. *Second Class*: Jones, Edward T., Int. Sc., University College Bangor; Fournier, d'Albe, E. E., Int. Sc., Normal School of Science and private study; Butters, Joseph B., Int. Sc., University College and private study; Lewis, Emily J., Int. Sc., Bedford College, London. *Third Class*: Wyatt, George H., Int. Sc., Normal School of Science and private study; Punnett, E. M., Int Sc., Mason College.

BOTANY.—*Second Class*: Tansley, Arthur G., Int. Sc., University College; Baldwin, Minnie, Int. Sc., Bedford College, London; Macdonald, E., Int. Sc., Girton College, Cambridge; Hayward, I. Mary, I. Sc., University College. *Third Class*: †Tafel, Albert, Prel. Sci., Owens College; Whipple, A. H., Int. Sc., St. John's College, Cambridge, and private tuition; Salter, Alfred, Prel. Sci., Guy's Hospital; Sanders, A. W., Prel. Sci., St. Mary's Hospital.

ZOOLOGY.—*First Class*: Davies, Arthur M., Int. Sc. (Exhibition), Normal School of Science, and private study. *Second Class*: ‡Blackman, S. S. F., Int. Sc., St. Bartholomew's Hospital; ‡Gillett, Henry T., Prel. Sci., St. Bartholomew's Hospital; Crouch, H. C., Prel. Sci., University College and Birkbeck Institution; †Toye, E. J., Prel. Sci., St. Bartholomew; C. Middle Class Schools and Birkbeck Institution. *Third Class*: †Gillies, Sinclair, Prel. Sci., St. Bartholomew's Hospital; ‡Lee, William Edward, Int. Sc., St. Bartholomew's Hospital; ‡Hampton, Thomas, Prel. Sci., St. Bartholomew's Hospital; Douglas, A. R. J., St. Bartholomew's Hospital; Ross, W. Mary, Int. Sc., University College, Aberystwith.

PRELIMINARY SCIENTIFIC (M.B.) EXAMINATION, JULY 1890.

*PASS LIST.*

ENTIRE EXAMINATION.—*First Division*: Allport, Wilfrid, King Edward High School and Mason College, Birmingham; Banting, Cecil, University College; Barchet, George Edward, London Hospital, and private study; Barron, Willie Netterville, Epsom College; †Best, John Duncan, School of Science and Art and Durham College School, Newcastle-on-Tyne; Blackman, Vernon Herbert, St. Bartholomew's Hospital; Brook, Sydney Wilkinson, Owens College; Chater, John Samuel, St. Bartholomew's Hospital; Clough, James Arthur, Yorkshire College; Collyer, Bertram Joseph, St. Bartholomew's Hospital; †Crace-Calvert, George Alfred, St. Bartholomew's Hospital; Crookshank, Francis Graham, University College; Currie, John, University College and private study; Dodgson, Robert William, Owens College; Fisk, Edward, University College; Ganner, Joseph, Mason College; Genge, George Gilbert, St. Thomas's Hospital; Griffiths, John Crisp, Mason College and private study; Hall, Joseph Percy, Owens College; †Handley, William Sampson, Guy's Hospital; Hibbert, Joseph Coote, University College; †Home, Alfred Lucette, St. Thomas's Hospital and Birkbeck Institution; Hunt, George Bertram, University College; Jacob, Frank Harwood, King's College; Melland, Charles Herbert, Owens College; Nolan, Harold, The Institute and University College, Liverpool; Norman, Richard Henry, St. Mary's Hospital; Paterson, William Henry John, St. Thomas's Hospital and Birkbeck Institution; †Pearson, Maurice Grey, St. Bartholomew's Hospital; Scowby, Edward Thomas, Guy's Hospital; Starkey, Thomas Albert, private study; Stebbings, John, B.A., Firth College and private study; Stevenson, Thomas Henry Craig, University College and Birkbeck Institution; Steward, Francis James, Guy's Hospital; Thompson, Henry Evans, St. Bartholomew's Hospital; Traill-Christie, Margaret M., University College; *Second Division*: Andrews, Henry Russell, London Hospital; Anning, F. Henry Elworthy, Epsom and University Colleges and private study; Benson, John Robinson, King's College; Bill, John Francis, St. Bartholomew's Hospital; Blakeley, Charles Swinnerton, Owen's College; †Brash, John Bardsley, University College; Brown, Joseph Norwood, University College; Brown, William C. T., B.A., Owen's College; Burnett, Frank Marsden, St. Bartholomew's Hospital; Coleman, Ernest, Guy's Hospital; Cunliffe, Thomas Varley, Owen's College; †Eder, Montague David, University Tutorial College and private study; Evans, Alfred Thomas, University College, Aberystwith; †Farrant, Mark, Westminster Hospital; Genge, George F. Saunders, Epsom College; Gibbons, Arthur Philip, London Hospital; Gregory, Thomas, Owen's College; Gullan, Archibald Gordon, University College, Liverpool; Hall, Edmund Stokes, Guy's Hospital; Hardcastle, William, Epsom College; Herbert, William, St. Thomas's Hospital and private study; Holliday, William Henry, Yorkshire College; †Horseman, James Walter, University College, Aberystwith; Horton, James Henry, Guy's Hospital; Hugo, James Henry, St. Bartholomew's Hospital; Hunt, Tom Harold, Owen's College; †Jeaffreson, Alfred Ernest, St. Paul's School; Jenkins, Arthur William, Epsom College; Johnston, Robert Macfie, Dulwich College; Kenrick, William Hamilton, private study; Knocker, William Douglas, St. Thomas's Hospital and Birkbeck Institution; McSorley, Alexander Somerled, King's College; Montgomery, Edwin Cecil, Epsom College; Moore, Eugene, Guy's Hospital; Phillips, John Evans, University College, Aberystwith; Raper, Matthew Henry, Epsom College; Bawsthorne, Hubert, University College; Read, Charles Stanford, University College and private tuition; Stork, Ernest Stanley, Royal College, Colombo, and University College; Strange, Robert Gordon, St. Paul's School and private study; Tribe, Paul C. Edward, King's College; Vellacott, Philip Northcott, Guy's Hospital; Wanhill, Charles Frederick, University College.

HONOURS CANDIDATES RECOMMENDED FOR A PASS. — Atkinson, William, B.A., University College and private study; †Barcroft, Joseph,

* Obtained the number of marks qualifying for the exhibition. † These candidates have also passed in the mathematics of the Intermediate examination in science, and have thus become admissible to the B.Sc. examination. ‡ § Denote equality of merit.

The Leys, Cambridge; Goffe, Ernest George Leopold, University College; *Richardson, Spencer William, private study and tuition.

Chemistry and Experimental Physics.—Bennett, William Boase, University College, Liverpool, and private study; Binckes, Frederick William, Dulwich College; Burman, Percy William, Firth College; Burrow, Lewis William, Queen's College, Taunton, and private study; Calverley, Joseph Ernest G., Dulwich College; Dobie, William Fullerton, The Medical School, Leeds; Evans, Evan Laming, Trinity College, Cambridge; Gordon, Cecil Frederick, Dulwich College; Griffiths, John A. Kendall, Epsom College; Hilton, Albert, Owen's College and private study: ‡Hunnard, Arthur, University College; ‡Kesteven, Percy Sandiland, St. Bartholomew's Hospital, University College, and private study; Moore, Julius, Guy's Hospital; Perry, Edmund Ludlow, St. Paul's School; Sanguinetti, Harold Herbert, University College; Savage, William George, University College; Scott, Herbert Ainslie, Owens College; Sellors, Thomas Blanchard, Mason College and private study; Skey, Arthur Richard Harrie, Dulwich College; Smellie, John Clementson, St. Mary's Hospital; Smith, Graham Udale, King's College; Smith, Percy Montague, Epsom College; Spon, Harry James, Guy's Hospital; Steinhæuser, John Robert, Guy's Hospital; Stickland, Cecil William, St. Paul's School; Temperley, Alfred John, London Hospital; Thomas, Thomas Morrell, University College, Aberystwith; Thurston, Edward Owen, private study; Tippett, Sydney Gordon, St. Mary's Hospital; ‡Webb, Mabel Elizabeth, Bedford College, London; Wells, Thomas Henry, private study; Williams, Horace Walter G., University College, Aberystwith.

Biology.—‡Adams, Percy Edward, St. Bartholomew's Hospital; Beadles, Arthur Harry, St. Bartholomew's Hospital and Birkbeck Institution; Bean, Algernon Carter, University College; Belfrage, Sydney Henning, University College and private study; Bergin, William Marmaduke, University College, Bristol; Beven, Octavius, Guy's Hospital; Blackwood, John, B.A., University College; Blake, Victor John, University College; ‡Brakenridge, Francis John, St. Thomas's Hospital; ‡Brebner, Arthur, University College; Bygott, Albert Henry, Lincoln School of Science and Mason College; ‡Cahill, John, University College and private study; Carter, Felix Bolton, University College; Cross, Walter Ernest, Guy's Hospital; Dimsey, Alfred, University College; Douglas, Stewart Ranken, St. Bartholomew's Hospital; Drury, Edward Guy Dru, University College; Dutton, William, Owens College; Earnshaw, Arthur, King's College and private study; Eddy, James, University College; Emms, Harry Robert, University College; ‡Erhardt, Conrad Charles James, King's College; Frazer, William Dyer, University College, Cardiff; Grant, Alexander Smeaton, London Hospital; Harnett, Charles John, Guy's Hospital; Hill, Reginald Augustus Lowder, St. Thomas's Hospital; ‡Hindley, Godfrey J. Douglas, Dulwich College; Hood, Thomas, St. Bartholomew's Hospital and Birkbeck Institution; Hopkins, Walter Kempson, St. Bartholomew's Hospital; ‡Horner, William Ernest Leethem, University College; Horwitch David, Mason College; ‡Hunt, Edward Lewis, University College; ‡Hutton, Albert Edward, Yorkshire College; ‡Jameson, Robert William, St. Bartholomew's Hospital; ‡Kitson, John Pole, University College; Lund, Kenneth Fraser, Owens College; Marks, Leonard Freeman, University College; Mason, Sydney Herbert, Mason College; Mather, Frederic Henry Vaughan, University College, Bristol; Miller, Walter Richard Samuel, University College, Bristol; ‡Miskin, Leonard John, St. Thomas's Hospital; Muir, Joseph Corbett, The Leys, Cambridge; Naish, Albert Ernest, University College, Bristol; Nicol, William Percy, King Edward High School and Mason College, Birmingham; Nunn, James Henry Francis, St. Bartholomew's Hospital; ‡Oliver, Helen Forsyth, University College and private tuition; Pedley, Arthur James, King Edward High School and Mason College, Birmingham; Phipps, John Hare, Owens College; Pike, Norman Howard, Dulwich College; ‡Price, George Basil, University College, Bristol; Rice, David, Guy's Hospital; Rusby, Edward Lionel M., King's College; Shardlow, Joseph, Firth College; ‡Shoppee, Sidney Edward, University College; Smith, Frank Addinsell, St. Bartholomew's Hospital; Smith, Lewis Albert, London Hospital; Spillane, James Charles, London Hospital; ‡Steen, Robert Hunter, private study and Queen's College, Belfast; Thomas, Leonard Kirkby, King Edward High School, and Mason College, Birmingham; Walker, Henry Roe, King's College; Waring, Anthony Henry, University College; Waymark, William Ebenezer, Guy's Hospital; Wilks, Morris, University College; Wise, Harry Mortimer, Guy's Hospital.

Two Subjects of the Examination (under former Regulations) †‡.—Bennett, R. Allan (C., B.) Mason College; Bostock, E. Bernard (P., B.) Mason College; Coutts, Francis James (P., B.) University College; Hunter, Clement H. (C., B.) Guy's Hospital and City School of Chemistry; Orton, K. Joseph P. (C., B.) St. Thomas's Hospital; Smith, Herbert Sidney (C., B.) Owens College; Thornton, Jeremiah (C., B.) Mason College and London Hospital; Townend, Richard H. (P., B.) London Hospital.

One Subject of the Examination (under former Regulations) †‡.—Morris, Edward (C.) St. Bartholomew's Hospital; Mumford, Wilfred G. (C.) Guy's Hospital; Rigby, Hugh Mallinson (C.) Dulwich and University Colleges, and London Hospital.

* These Candidates have also passed in the Mathematics of the Intermediate Examination in Science, and have thus become admissible to the B.Sc. Examination.

† The subjects taken up by these Candidates are indicated by initials after the names—C.=Chemistry; P.=Physics; B.=Biology.

‡ These Candidates have now completed the Examination.

[N.B.—The names of Candidates who have obtained honours do not appear in this list.]

### Scraps and Gleanings.

Dr. Tuzo, an American, dropped down dead at Warlingham last week.

With reference to the paragraph in this column, last week, on the Croydon Infirmary, we have much pleasure in stating, on the authority of the medical superintendent, that the nurses were not in the slightest degree concerned in the matters referred to. He requests us, therefore, to withdraw the remarks so far as the nurses are concerned, which we have much pleasure in doing.

